# Transcriptional activation of follistatin by Nrf2 protects pulmonary epithelial cells against silica nanoparticle-induced oxidative stress

**DOI:** 10.1038/srep21133

**Published:** 2016-02-16

**Authors:** Chen Lin, Xinyuan Zhao, Desen Sun, Lingda Zhang, Wenpan Fang, Tingjia Zhu, Qiang Wang, Botao Liu, Saisai Wei, Guangdi Chen, Zhengping Xu, Xiangwei Gao

**Affiliations:** 1 Institute of Environmental Medicine, Zhejiang University School of Medicine, Hangzhou, China; 2 Collaborative Innovation Center for Diagnosis and Treatment of Infectious Diseases, Zhejiang University, Hangzhou, China; 3 Program in Molecular Cell Biology, Zhejiang University School of Medicine, Hangzhou, China; 4 University of Massachusetts Medical School, Program in Molecular Medicine, Worcester, MA, 01605, USA

## Abstract

Silica nanoparticles (SiO_2_ NPs) cause oxidative stress in respiratory system. Meanwhile, human cells launch adaptive responses to overcome SiO_2_ NP toxicity. However, besides a few examples, the regulation of SiO_2_ NP-responsive proteins and their functions in SiO_2_ NP response remain largely unknown. In this study, we demonstrated that SiO_2_ NP induced the expression of follistatin (FST), a stress responsive gene, in mouse lung tissue as well as in human lung epithelial cells (A549). The levels of Ac-H3(K9/18) and H3K4me2, two active gene markers, at FST promoter region were significantly increased during SiO_2_ NP treatment. The induction of FST transcription was mediated by the nuclear factor erythroid 2-related factor 2 (Nrf2), as evidenced by the decreased FST expression in Nrf2-deficient cells and the direct binding of Nrf2 to FST promoter region. Down-regulation of FST promoted SiO_2_ NP-induced apoptosis both in cultured cells and in mouse lung tissue. Furthermore, knockdown of FST increased while overexpression of FST decreased the expression level of NADPH oxidase 1 (NOX1) and NOX5 as well as the production of cellular reactive oxygen species (ROS). Taken together, these findings demonstrated a protective role of FST in SiO_2_ NP-induced oxidative stress and shed light on the interaction between SiO_2_ NPs and biological systems.

Nanoparticles (NPs) are engineered structures with less than 100 nanometers in at least one dimension. These materials are increasingly being used for diverse industrial and biomedical applications as well as in consumer products^1^. The wide use of NPs has raised serious concerns about their safety for human health and the environment. In addition, NPs are different from large particles in physicochemical properties and may potentially result in yet unknown toxic effects^2^,^3^. Among the engineered nanomaterials, silica nanoparticle (SiO_2_ NP) is one of the most widely applied^4^. Despite intense investigations, current knowledge of the physiological effects of SiO_2_ NPs on biological barriers and the underlying molecular mechanisms remains fragmented.

The respiratory system is considered to be one of the main routes by which NPs access human body^1^,^2^,^3^. Therefore, the respiratory toxicity of NPs draws great concern. In response to NP exposure, airway epithelial cells and macrophages generate reactive oxygen species (ROS) by biologically catalyzed redox reactions. ROS damages cellular proteins, lipids, and DNA^1^,^5^. On the other hand, elevated ROS triggers cellular oxidative stress responses which are mainly mediated by the activation of nuclear factor erythroid 2-related factor 2 (Nrf2) signaling^6^. Nrf2 is a transcription factor which is rapidly degraded under normal condition by ubiquitin-proteasome system. During oxidative stress, the protein is stabilized, translocates into the nucleus and thereby induces the expression of protective proteins including antioxidant enzymes, detoxification enzymes, and stress-response proteins^6^,^7^. These NP-responsive proteins exert antioxidant and cytoprotective effects in lungs. If the protection effect fails, escalation of oxidative stress results in mitochondrial perturbation and cell apoptosis^1^,^7^,^8^. However, besides a few Nrf2 target genes, the regulation of other SiO_2_ NP-responsive genes and their functions in SiO_2_ NP response remain largely unknown.

Follistatin (FST) is widely expressed in higher animals and participates in a variety of processes such as cell growth, development, differentiation, and secretion^9^. FST was firstly identified as a secretory protein that binds and inactivates transforming growth factor (TGF)-β family members including activin, bone morphogenetic proteins (BMPs), and myostatin. The TGF-β-like molecule-neutralizing effect of FST contributes to most of its functions^10^,^11^,^12^. Recent studies showed that FST also plays a protective role under cellular stresses including oxidative stress^13^,^14^,^15^. For example, activin induces endothelial cell oxidative stress and endothelial cell dysfunction, and these effects are mitigated by follistatin^15^. Follistatin has also been reported to suppress BMP4-induced ROS production^16^. Based on the inhibitory role of FST in cellular ROS production, we proposed that FST participates in cellular response to SiO_2_ NP-induced oxidative stress.

In this study, we evaluated the function of FST in SiO_2_ NP-induced lung toxicity using both *in vitro* and *in vivo* models. We found that FST was transcriptionally up-regulated by transcription factor Nrf2 during SiO_2_ NP treatment. Our data further demonstrated that FST protected cells against apoptosis through reversing oxidative stress. These data provided evidence to support that FST is an oxidative stress responsive protein contributing to lung anti-oxidant response to SiO_2_ NPs.

## Results

### Characterization of SiO_2_ NPs

**Figure 1:**
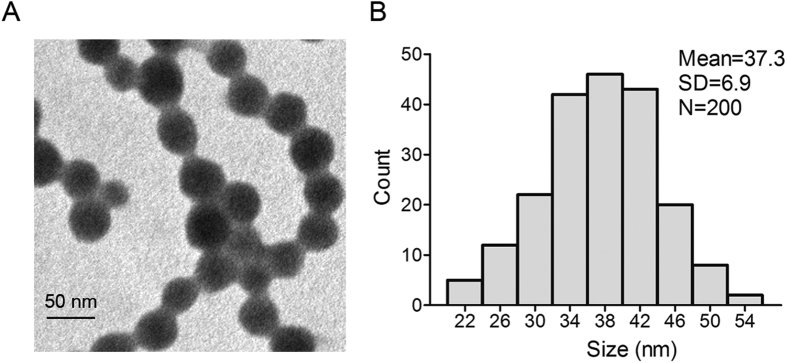
Characterization of silica nanoparticles. (**A**) Transmission electron microscopy image of SiO_2_ NPs. (**B**) Size distribution of SiO_2_ NPs. Silica nanoparticles exhibited good monodispersity and showed approximately normal distribution.

We first evaluated the size of silica nanoparticles using TEM. Data showed that the silica nanoparticles had a spherical shape with the average diameter of 37.3 nm (Fig. 1A). Size measurement by Image J software showed that SiO_2_ NPs were approximately normally distributed (Fig. 1B). The hydrodynamic size of silica nanoparticles in culture media RPMI-1640 was 41.36 ± 2.55 nm and the silica nanoparticles exhibited good monodispersity. SiO_2_ NPs are quite stable in RPMI 1640 media since the size did not increase after 24 h of incubation (41.32 ± 3.19 nm, Table 1). Further, we measured zeta potentials of SiO_2_ NPs, which provide quantitative information on the stability of the particles. Data showed that the absolute value of zeta potentials of SiO_2_ NPs in our study was −33.28 ± 0.65 and −35.14 ± 0.71 mV respectively, indicating that the particles are more likely to remain dispersed.

### SiO_2_ NP enhances FST expression in mouse lung tissue

**Figure 2:**
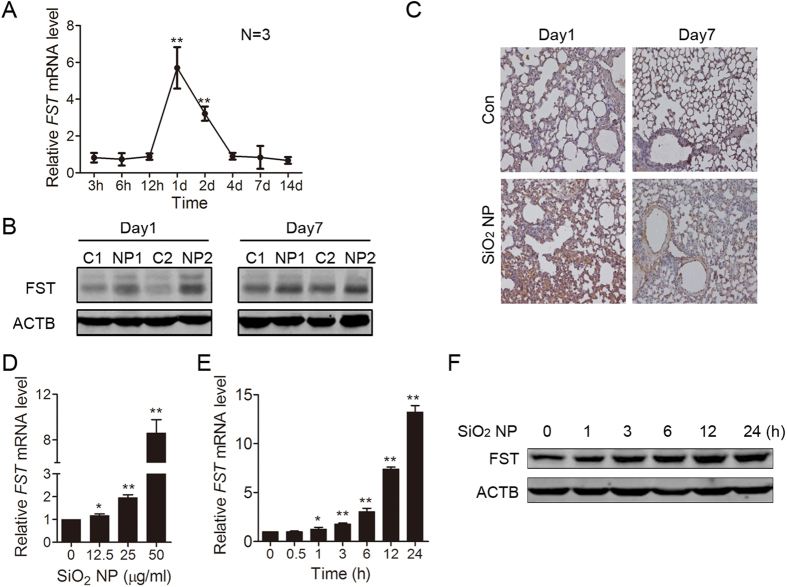
SiO_2_ NP enhances FST expression in mouse lung tissue and in A549 cells. (**A–C**) Mouse lung was instilled with PBS or 100 μg of SiO_2_ NPs and harvested at the indicated time. (**A**) The FST mRNA level in mouse lung tissue was measured by real time qPCR and normalized to *ACTB* mRNA level. (**B**) The lung tissue protein lysates from paired mice were subjected to immunoblotting for FST detection. Two mice were used for each group (C: PBS; NP: SiO_2_ NP). (**C**) Fixed lung tissues from paired mice were subjected to immunohistochemistry with FST antibody. (**D**) A549 cells were incubated with SiO_2_ NPs at different concentrations for 12 h. The FST mRNA level was measured by real time qPCR. Data are presented as the mean ± SD of three independent experiments. *P < 0.05, **P < 0.01. (**E**,**F**) A549 cells were incubated with or without 50 μg/ml of SiO_2_ NPs and harvested at the indicated time. (**E**) The FST mRNA level was measured by real time qPCR. Data are presented as the mean ± SD of three independent experiments. *P < 0.05, **P < 0.01. (**F**) The FST protein level was detected by immunoblotting.

FST is up-regulated during glucose deprivation and plays a protective role^13^,^14^. We hypothesized that FST is also a SiO_2_ NP-responsive protein. Since lung is the major exposure tissue for SiO_2_ NPs, we detected whether SiO_2_ NP treatment could induce FST expression change in mouse lung tissue. To that end, we intratracheally instilled mice with SiO_2_ NPs. The mRNA level of FST increased (5.7 fold) at 1 day after SiO_2_ NP instillation and decreased to the basal level 4 days after SiO_2_ NP treatment (Fig. 2A, Fig. S1). Immunoblotting showed that the protein level of FST significantly increased at 1 day after SiO_2_ NP treatment, while the increase of FST protein at 7 days was not as dramatic as 1 day (Fig. 2B). Furthermore, immunohistochemistry staining showed increased expression of FST in SiO_2_ NP-treated lung tissue (Fig. 2C). These data indicated that FST is an early responsive gene to SiO_2_ NP treatment.

### SiO_2_ NP enhances FST expression in A549 cells

Epithelial cells are the major components of lung tissue and essential for lung function. We asked whether the expression of FST in lung epithelial cells increased during SiO_2_ NP treatment. Thus, we treated human epithelial cells A549 with SiO_2_ NPs and detected FST mRNA level. Data showed that SiO_2_ NP up-regulated FST mRNA level in a dose-dependent manner. 50 μg/ml of SiO_2_ NP treatment induced 8-fold increase of FST mRNA (Fig. 2D). The mRNA level of FST started to increase 1 hour after SiO_2_ NP treatment, while the protein level started to increase at 3 hours of SiO_2_ NP treatment. The expression of FST reached the peak at 24 hours (Fig. 2E,F). These data indicated that SiO_2_ NP promotes FST expression in lung epithelial cells. We also detected the effect of SiO2 NP in normal branchial epithelial cell line BEAS-2B. Data showed that SiO2 NP also promoted FST expression in BEAS-2B cells (Fig. S2).

### The transcription of FST gene is activated during SiO_2_ NP treatment

**Figure 3:**
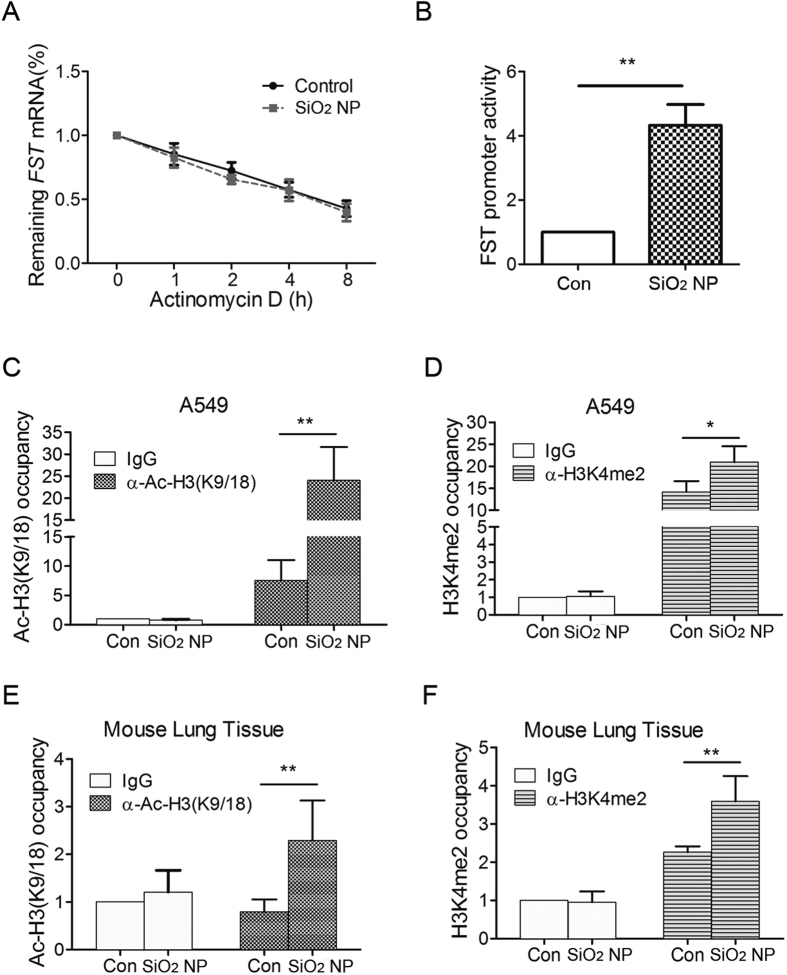
The transcription of FST gene is activated during SiO_2_ NP treatment. (**A**) A549 cells treated with or without SiO_2_ NPs were incubated with 5 μg/ml of actinomycin D for 1, 2, 4 and 8 h. FST mRNA level was measured and normalized to *ACTB* mRNA level. (**B**) A549 cells transfected with FST promoter luciferase reporter and renilla luciferase internal control were treated with or without 50 μg/ml of SiO_2_ NPs for 12 h. Luciferase activity was then detected and normalized to renilla activity. (**C,D**) A549 cells were treated with or without 50 μg/ml of SiO_2_ NPs for 12 h. ChIP analysis was performed with antibodies against Ac-H3(9/18) (**C**) or H3K4me2 (**D**) and analyzed by qPCR. (**E,F**) Mouse lung was instilled with PBS or 100 μg of SiO_2_ NPs for 1 day. ChIP analysis was performed with antibodies against Ac-H3(9/18) (**E**) or H3K4me2 (**F**) and analyzed by qPCR. The occupancies of Ac-H3(9/18) or H3K4me2 at *FST* promoter region were normalized to *ACTB*. The values are the mean ± SD of three independent experiments. *P < 0.05, **P < 0.01.

The higher level of FST mRNA may result from either transcriptional activation or post-transcriptional regulation^14^. We first examined the stability of FST mRNA. Actinomycin D was used to block *de novo* RNA synthesis, and then the persistence of the existing FST mRNA was measured by quantitative RT-qPCR at 1, 2, 4 and 8 h. Our results revealed that the stability of the FST mRNA did not change during SiO_2_ NP treatment (Fig. 3A). Actually, when *de novo* RNA synthesis was inhibited, *FST* mRNA level could not increase during SiO_2_ NP treatment (Fig. S3), suggesting that SiO_2_ NP regulates *FST* mRNA expression at transcriptional level. Therefore, we detected changes in FST gene promoter activity using a luciferase reporter. Data showed that the transcriptional activity of FST promoter region was significantly induced under SiO_2_ NP treatment (Fig. 3B). Since Ac-H3(K9/18) and H3K4me2 are recognized as active gene markers^17^, we further detected the Ac-H3(K9/18) and H3K4me2 levels at *FST* promoter region using chromatin immunoprecipitation. Data showed that SiO_2_ NP significantly increased both Ac-H3(K9/18) and H3K4me2 levels at *FST* promoter region (Fig. 3C,D). Ac-H3(K9/18) and H3K4me2 levels at *FST* promoter also increased in SiO_2_ NP-treated mouse lung tissue (Fig. 3E,F), further supporting that SiO_2_ NP up-regulates FST expression at the transcriptional level.

### Nrf2 mediates FST induction during SiO_2_ NP treatment

**Figure 4:**
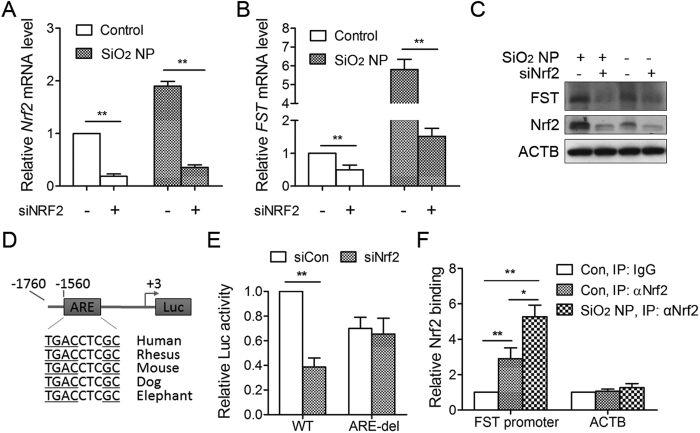
Nrf2 mediates FST induction during SiO_2_ NP treatment. (**A–C**) A549 cells transfected with siRNA targeting Nrf2 (siNrf2) or control siRNA were incubated with or without 50 μg/ml of SiO_2_ NPs for another 12 h. The mRNA level of Nrf2 (A) and FST (**B**) was detected by qPCR and the protein level was detected by immunoblotting (**C**,**D**) Schematic of a luciferase reporter of the human FST promoter containing an evolutionarily conserved ARE among vertebrate species. (**E**) A549 cells transfected with siRNA targeting Nrf2 or control siRNA together with wild-type or ARE-deleted FST promoter luciferase reporter construct. Luciferase activity was measured 24 h following plasmid transfection. (**F**) A549 cells were treated with or without 50 μg/ml of SiO_2_ NPs for 12 h. ChIP analysis was performed with antibodies against Nrf2 and analyzed by qPCR. The values are the mean ± SD of three independent experiments. *P < 0.05, **P < 0.01 (One way ANOVO).

The transcriptional factor Nrf2 controls stress gene expression during SiO_2_ NP treatment^6^,^7^. To determine the role of endogenous Nrf2 in regulating FST expression, Nrf2 levels was decreased by siRNA (Fig. 4A,C). Knockdown of Nrf2 resulted in significant reduction of FST mRNA and protein expression under normal condition and SiO_2_ NP treatment (Fig. 4B,C), indicating that FST transcription is under the control of Nrf2.

By analyzing the FST gene proximal promoter, we identified a putative antioxidant response element (ARE), which is a consensus Nrf2-binding sequence (Fig. 4D). To determine the direct regulation of Nrf2 on FST expression, the luciferase reporters containing FST promoters with either wild-type or ARE-deleted sequences were used. Data revealed that luciferase activity of the wild-type FST promoter reporter was significantly reduced (to 40% of control levels) following knockdown of Nrf2, indicating that FST promoter activity is Nrf2-dependent. However, deletion of ARE region completely abolished Nrf2 effect (Fig. 4E).

Finally, we sought to determine whether Nrf2 can directly bind to the FST promoter using ChIP assays. Data unveiled enrichment of FST promoter region (2.9 fold) but not ACTB gene following immunoprecipitation with the Nrf2 antibody. SiO_2_ NP treatment significantly increased Nrf2 binding (5.3 fold) to FST promoter (Fig. 4F). These data indicated that *FST* gene is a direct target of Nrf2.

### FST protects against SiO_2_ NP-induced cell apoptosis

**Figure 5:**
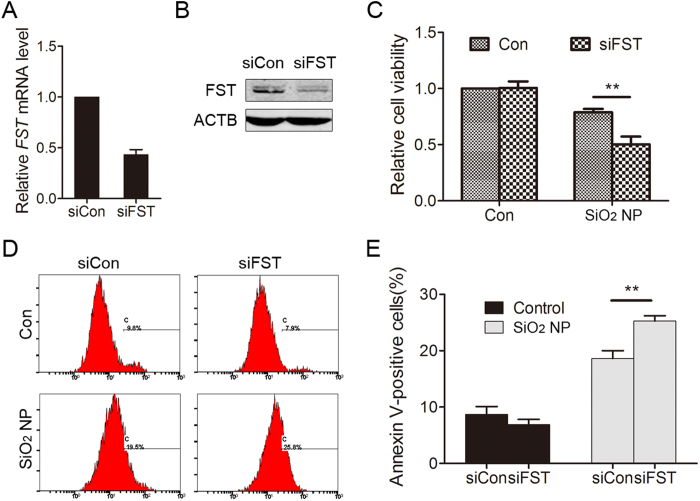
FST protects against SiO_2_ NP-induced cell apoptosis. (**A,B**) A549 cells were transfected with siRNA targeting FST (siFST) or control siRNA (siCon). The *FST* mRNA level was detected by qPCR (**A**) and the FST protein level was detected by immunoblotting (**B–E**) A549 cells transfected with siRNA targeting FST (siFST) or control siRNA (siCon) were treated with or without 50 μg/ml of SiO_2_ NPs for 12 h. (**C**) Cell viability was determined using cell counting kit-8 (CCK-8). (**D**) Cell apoptosis was monitored by annexin V-FITC staining and flow cytometry analysis. (**E**) Annexin-V positive cells were quantified. The values are the mean ± SD of three independent experiments. **P < 0.01.

SiO_2_ NP treatment induces cellular toxicity both *in vitro* and *in vivo*^1^,^18^,^19^. Since FST expression was up-regulated in response to SiO_2_ NP, we suspected that FST has a protective role. To test this hypothesis, the expression of FST was down-regulated using siRNA (Fig. 5A,B) and cell viability was detected. Cell viability significantly decreased in FST silenced cells compared to control cells after SiO_2_ NP treatment (Fig. 5C). Decreased cell viability might be due to cell apoptosis. We therefore determined cellular apoptosis by annexin V-FITC staining method. The fractions of annexin V-positive cells were low in control groups (8.7 ± 1.4%, and 6.9 ± 0.9% respectively). SiO_2_ NP treatment markedly increased cell apoptosis in control siRNA-transfected cells (18.6 ± 1.4%), and elimination of FST enhanced the apoptotic tendency to 25.3 ± 1.0% (*P* < 0.01 compared to control) (Fig. 5D,E), indicating FST has a protective role in apoptosis.

### FST protects against SiO_2_ NP toxicity *in vivo*

**Figure 6:**
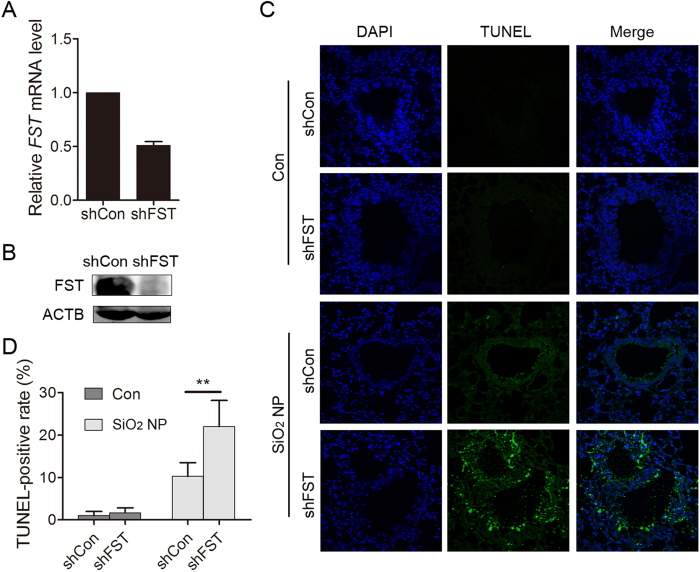
FST protects against SiO_2_ NP toxicity *in vivo*. (**A,B**) Mouse embryo fibroblast cells were infected with viruses expressing shCon or shFST. The *FST* mRNA level was detected by qPCR (**A**) and the FST protein level was detected by immunoblotting (**B–D**) Mouse lung infected with shCon or shFST was instilled with PBS or 100 μg of SiO_2_ NPs for 1 day. (**C**) Cell apoptosis was detected by TUNEL assay. (**D**) TUNEL positive cells were quantified. The values are the mean ± SD. **P < 0.01.

Next, we asked whether FST protects against SiO_2_ NP toxicity in lung tissue. To that end, the FST expression was down-regulated using virus-mediated shRNA and mice were treated with SiO_2_ NPs for 1 day. Cell apoptosis was then determined by TUNEL assays. Both qPCR and immunoblotting data showed that shRNA significantly reduced FST expression in mouse cells (Fig. 6A,B). Almost no TUNEL-positive cells were observed in the control group, while obvious TUNEL-positive staining was observed in SiO_2_ NP-treated lungs, indicating that SiO_2_ NP exposure induced cell apoptosis in lung tissue^18^,^20^. The number of TUNEL-positive cells increased in FST-KD group (Fig. 6C,D), indicating that FST protects against SiO_2_ NP toxicity *in vivo*.

### FST inhibits SiO_2_ NP-induced ROS production and the expression of NOX1 and NOX5 genes

**Figure 7:**
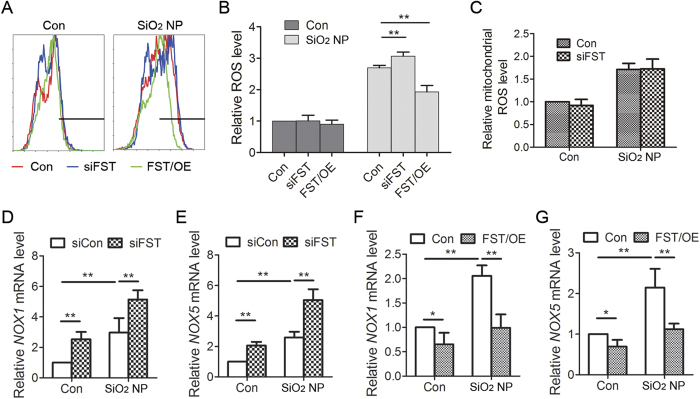
FST inhibits SiO_2_ NP-induced ROS production. (**A**) A549 cells transfected with siRNA targeting FST (siFST) or FST gene (FST/OE) were treated with or without 50 μg/ml of SiO_2_ NPs for 12 h. Intracellular ROS generation was detected by DCFH-DA fluorescent probe and flow cytometry. (**B**) Relative ROS level was calculated. The values are the mean ± SD of three independent experiments. **P < 0.01. (**C**) A549 cells transfected with siRNA targeting FST (siFST) were treated with or without SiO_2_ NPs for 12 h. Mitochondrial ROS was detected using MitoSOX. (**D,E**) A549 cells transfected with control siRNA or siRNA targeting FST (siFST) were treated with or without 50 μg/ml of SiO_2_ NPs for 12 h. The mRNA level of NOX1 (**D**) and NOX5 (**E**) was measured by real time qPCR. (**F,G**) A549 cells overexpressing FST gene were treated with or without 50 μg/ml of SiO_2_ NPs for 12 h. The mRNA level of NOX1 (**F**) and NOX5 (**G**) was measured by real time qPCR. The data are presented as the mean ± SD of three independent experiments. **P < 0.01.

The toxic effect of SiO_2_ NPs is mainly mediated by particle-induced oxidative stress^1^. We therefore determined whether FST exerts its protective function through inhibiting ROS production during SiO_2_ NP exposure. SiO_2_ NP treatment increased total ROS level in A549 cells. Knockdown of FST further increased ROS level while overexpression of FST decreased ROS level (Fig. 7A,B), indicating an inhibitory role of FST in SiO_2_ NP-induced ROS production.

ROS is produced in mitochondria and by cytosolic NADPH oxidases (NOXs)^21^,^22^. Therefore, FST might inhibit ROS level by regulating either mitochondria or NOX functions. We firstly detected mitochondrial ROS level using MitoSOX assay and data showed that knockdown of FST did not affect mitochondrial ROS production (Fig. 7C). Next, we detected the expression of NOX1, NOX2, NOX3, NOX4, and NOX5, which are involved in cytosolic ROS production^21^. Our data showed that the mRNA levels of NOX2, NOX3 and NOX4 were very low in A549 cells. The mRNA levels of NOX1 and NOX5 significantly increased during SiO_2_ NP treatment, indicating that NOX1 and NOX5 are involved in SiO_2_ NP-induced cytosolic ROS synthesis. Knockdown of FST increased while overexpression of FST decreased the expression of NOX1 and NOX5 under both control and SiO_2_ NP treatment (Fig. 7D–G). These data suggested that FST inhibits NOX1 and NOX5 expression to regulate cytosolic ROS production.

## Discussion

**Figure 8:**
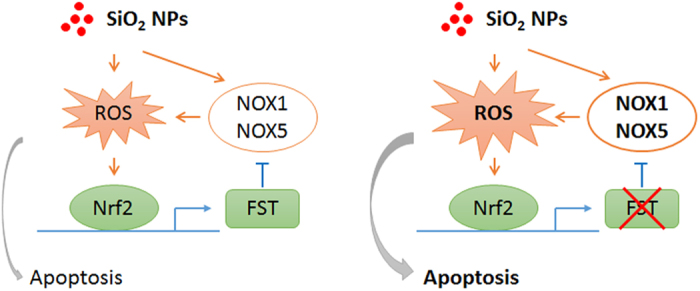
Schematic model for the role of FST in protecting against SiO_2_ NP toxicity. SiO_2_ NPs induce cellular ROS production through multiple pathways including the activation of NOX1 and NOX5. The increased cellular ROS activates the transcription factor Nrf2 which induces the transcription of FST. FST further inhibits the expression of NOX1 and NOX5. This negative feedback loop maintains cellular ROS homeostasis during SiO_2_ NP exposure (left panel). Elimination of FST breaks the feedback loop which increases cellular ROS level and induces cell apoptosis (right panel).

The toxicity of SiO_2_ NPs has gained much attention with the fast growth of nanotechnology. Both *in vitro* and *in vivo* evidence has shown that SiO_2_ NPs induce toxic effect in respiratory system^1^,^18^,^19^. However, our understanding towards cellular responses to overcome the SiO_2_ NPs toxicity is still limited. In this report, we provided experimental evidence to support that FST is involved in cellular protective response to SiO_2_ NPs. The major findings of our study include: 1) SiO_2_ NPs induced endogenous expression of FST both in mouse lungs and in cultured lung epithelial cells; 2) SiO_2_ NPs activated FST transcription through Nrf2; 3) FST inhibited SiO_2_ NP-induced apoptosis; 4) FST inhibited ROS production triggered by SiO_2_ NPs. These findings demonstrated a protective role of FST (Fig. 8) and might provide a better understanding of the interaction between SiO_2_ NPs and biological systems.

FST is a stress responsive gene and its expression can be induced by SiO_2_ NP treatment as well as glucose deprivation. However, the mechanism is totally different. We have shown that the induction of FST by glucose deprivation was due to an increase in the half-life of its mRNA. Under normal condition, AUF1 bound to AU-rich element in the 3’UTR of FST mRNA and promoted FST mRNA decay. During glucose deprivation, a majority of AUF1 shuttled from cytoplasm to nucleus, resulting in dissociation of AUF1 from FST mRNA and thus stabilization of FST mRNA^14^. However, our present data showed that the half-life of FST mRNA did not change during SiO_2_ NP-treatment. On the contrary, the Ac-H3(K9/18) level and H3K4me2 level at FST promoter region significantly increased, indicating an activation of FST transcription. Therefore, SiO_2_ NP regulates FST expression at transcriptional level while glucose deprivation regulates FST expression at post-transcriptional level. This might be due to the different signaling activated. Glucose deprivation activates AMPK pathway and AMPK activation mediates FST induction during glucose deprivation as AMPK inhibition blocked the increase of FST mRNA^14^. On the contrary, SiO_2_ NPs mainly induce ROS generation and oxidative stress. SiO_2_ NP-induced ROS production activates transcription factor Nrf2, which induces FST transcription. This was supported by the decreased FST expression in Nrf2-deficient cells and the direct binding of Nrf2 to FST promoter region (Fig. 4).

How does FST exert its protective function during SiO_2_ NP treatment? NP-induced ROS generation and oxidative stress is the best-developed paradigm to explain the cellular responses to nanoparticles. Our data demonstrated that FST inhibited NOX1 and NOX5 expression and ROS production, indicating that the protective effect of FST is due to the ROS-inhibition. Although the detailed mechanism by which FST inhibits ROS production is still unclear, there are clues pointing to the TGF-β-like molecule-neutralizing effect of FST. It has been reported that TGF-beta family members increased cellular ROS level in a variety of cell lines while FST antagonized their effects. For example, myostatin induced oxidative stress by producing ROS in skeletal muscle cells^23^. Activin induced endothelial cell oxidative stress and endothelial cell dysfunction, and these effects are mitigated by follistatin^15^. BMP4 treatment increased ROS levels in basal progenitor cells, while follistatin inhibited BMP4-induced ROS production^16^. All these data support that FST is a negative regulator of cellular ROS production.

Nanoparticles were reported to induce general toxic effects on cells such as oxidative stress, inflammation and cell death. However, different NPs have different physic-chemical properties, they might induce NP-specific cytotoxicity. For example, a panel of industrially most relevant metal oxide nanoparticles was screened for toxic effects in A549 cells. Data showed that SiO_2_ NPs induced acute cytotoxicity, which is consistent with our results. SiO_2_ NPs also induced the expression of COX-2, TNF-α, IL-1β, IL-6 and IL-8. However, iron oxide- or titania- NPs were not cytotoxic and only specifically induced TNF-α expression^24^. Due to the different cytotoxicity, the following cellular protective responses might also be different. Shi J *et al.* reported that microsomal glutathione transferase 1 (MGST1) protected against SiO2 nanoparticle-induced oxidative stress and cytotoxicity, while it failed to prevent ZnO nanoparticle-triggered cell death^25^. Our data showed that FST can also be induced by TiO_2_ NPs, Ag NPs, and ZnO NPs, but the expression level of FST did not change significantly under gold nanorods (GNRs) or graphene oxide (GO) treatment (Fig. S4). These data indicated the existence of different mechanism of cytotoxicity of different nanoparticles.

In summary, we have identified FST as a SiO_2_ NP-responsive protein that protects against SiO_2_ NP lung toxicity. These findings point to a protective strategy for respiratory system to overcome SiO_2_ NP-induced adverse effects.

## Materials and Methods

### Silica nanoparticles

SiO_2_ nanoparticle was from Sigma-Aldrich (#637238, Sigma-Aldrich, St. Louis, MO, United States). Nanoparticles were dispersed in double distilled water at the concentration of 10 mg/mL. Nanoparticles were sonicated at 200 W for 30 seconds prior to cell or mouse treatment.

Transmission electron microscope (TEM) was taken by a JEOL JEM-1200EX transmission electron microscope for nanoparticles. SiO_2_ NPs were dispersed in Roswell Park Memorial Institute (RPMI)-1640 medium (Invitrogen, Carlsbad, CA, United States) for 0 h and 24 h and then subjected to dynamic light scattering (DLS) and zeta-potential measurements using the instrument Zetasizer Nano ZS-90 (Malvern Instruments, Orsay, France).

### Cells culture and nanoparticle treatment

Lung epithelial cells (A549) were obtained from ATCC and cultured in RPMI-1640 medium supplemented with 10% fetal bovine serum (Thermo Fisher Scientific, Waltham, MA, United States). Cells were maintained at 37 °C in an atmosphere containing 5% CO2 and 100% humidity.

For SiO_2_ NP treatment, cells were seeded in 6-well plates. After 24 hours, cells were treated with silica nanoparticles suspended in RPMI-1640 supplemented with 1% fetal bovine serum at different concentration for indicated time and then subjected to further experiments.

### Plasmids

Plasmid expressing FST288 was kindly provided by Dr. Henry T. Keutmann (Massachusetts General Hospital).

For the construction of FST promoter plasmid, FST promoter region was amplified by PCR and cloned to vector pGL3-basic between *Xho*I and *Hind*III sites. To construct Nrf2-binding site deletion mutant, the 5’ and 3’ parts of FST promoter region was amplified and ligated using *Bam*HI site.

For the construction of shRNA plasmid targeting FST, lentviral vector pLVX-shRNA1 was used. shRNA oligonucleotides were designed using online protocol www.clontech.com. Oligo sequences were listed in supplemental Table 1. Synthesized DNA oligos were annealed and ligated into the *Bam*HI/*Eco*RI-digested pLVX-shRNA1 vector.

### RNA purification and reverse transcription reaction

Total RNA was isolated with Trizol reagent (Invitrogen) following the manufacturer’s protocol. 0.5 μg of total RNA was reverse transcribed using random hexamers and the High Capacity cDNA Reverse Transcription Kit (Life Technologies, Grand Island, NY, United States).

### Real-time quantitative PCR analysis

Real-time quantitative PCR analysis was performed in 10-μl reactions using SYBR GREEN PCR Master Mix (Applied Biosystems). The related mRNA level was normalized to the *β-actin* mRNA level. Data were analyzed using the 2^−ΔΔCt^ method^26^. Sequences of all the primers used for PCR amplification are listed in supplemental Table 1.

### Immunoblotting analysis

Proteins were quantified by BCA protein assay kit (Bio-Rad) and applied to immunoblotting analysis as described previously [2]. 50 μg of total proteins were subjected to sodium dodecylsulfate polyacrylamide gel electrophoresis (SDS-PAGE) and transferred to nitrocellulose membrane (Whatman, Clifton, NJ, United States). Membrane was blocked with 3% bovine serum albumin (BSA) TBS-T buffer (20 mM Tris-HCl, pH 8.0, 150 mM NaCl, 0.05% Tween- 20), probed with antibodies targeting to FST (Santa Cruz Biotechnology, Santa Cruz, CA, United States), or ACTB (Cell Signaling Technology, Beverly, MA, United States). Membrane was incubated with horseradish-conjugated secondary antibodies, detected with the SuperSignal West Pico chemiluminescence substrate (Thermo Fisher Scientific), and finally exposed to an X-ray film. Alternatively, membrane was incubated with fluorescent secondary antibodies, and visualized by an Odyssey imaging system (Li-COR Biosciences, Lincoln, NE, United States).

### Immunohistochemistry

Embedded mouse lung tissues were deparaffinized with xylene, rehydrated in ethanol, and boiled in 10 mM citrate buffer (pH 6.0) for 30 min for antigen retrieval. Endogenous peroxidase was blocked by treatment with 3% H_2_O_2_. After blocking in goat serum for 30 min at room temperature, tissues were incubated with anti-FST (Santa Cruz Biotechnology) at 4 °C overnight. The slides were then visualized with Envision System (DAKO Corporation, Carpinteria, CA, United States) and counterstained with hematoxylin. Images were captured with a wide field microscope (Nikon Eclipse Ti, Nikon Instruments Inc., Shanghai, China).

### RNA interference

For knockdown experiments, A549 cells were transiently transfected with 100 pmol of the chemically synthesized siRNAs targeting FST or the negative control siRNA using LipofectamineRNAiMAX (Invitrogen) following the manufacturer’s recommendations. Cells were harvested 48 hours post-transfection. siRNAs were synthesized by GenePhama company (Shanghai, China). siRNA sequences used are designed as follows: FST siRNA, forward, GAUCUAUUGGAUUAGCCUATT, reverse, UAGGCUAAUCCAAUAGAUCTT; Nrf2 siRNA, forward, GAAUGGUCCUAAAACACCATT, reverse, UGGUGUUUUAGGACCAUUCTT; negative control siRNA, forward, UUCUCCGAACGUGUCACGUTT, reverse, ACGUGACACGUUCGGAGAATT.

### Luciferase assay

A549 cells were transfected with FST promoter and pRL-TK was co-transfected as the internal control. 24 hours after transfection, cells were treated with SiO_2_ NPs for another 12 hours. Cells were then lysed and luciferase activity was measured by the Dual-Luciferase assay system (Promega, Madison, WI, United States). The firefly luciferase activity was normalized to renilla luciferase.

### ROS detection

Total ROS was detected by using a fluorescent probe 2’,7’-dichlorofluorescin-diacetate (DCFH-DA) (Beyotime, Nanjing, China). Briefly, cultured A549 cells were treated with or without SiO_2_ NPs for 12 h and then incubated with 10 μM of DCFH-DA at 37 °C for 30 minutes. After rinsing with PBS to eliminate excess DCFH-DA, cells were harvested in 0.5 ml PBS and the fluorescence intensity was monitored with flow cytometry (FCM).

MitoSOX (Invitrogen) was used to detect mitochondrial ROS. Cultured A549 cells were collected and incubated with 10 μM of MitoSOX at 37 °C for 30 minutes and the fluorescence intensity was monitored with flow cytometry.

### Apoptosis assay

Cells seeded in 6-well plates were transfected with siRNAs or plasmids for 48 hours. After SiO_2_ NP treatment for 12 hours, both floating and attached cells were harvested and applied to annexin V detection. Briefly, resuspended cells were stained with 5 μl of annexin V-FITC (Biovision, Mountain View, CA, United States) for 5 minutes in the dark and then analyzed by flow cytometry (Beckman Coulter, Brea, CA, United States).

### CCK8-based viability assay

The effect of SiO_2_ NP on cell viability was assessed by Cell Counting Kit-8 (CCK8) assay (Dojindo Laboratory, Japan). A549 cells plated in 96-well plate were transfected with siRNAs for 48 hours. Cells were then treated with SiO_2_ NPs for another 12 hours. 10 μl of CCK8 reagent was added to each well and the cells were incubated for 2 hours at 37 °C. The optical density (OD) at 450 nm was measured by using *Varioskan*Flash (Thermo Scientific).

### shRNA plasmid construction

To produce lentivirus expressing FST-shRNA, the plasmid pLVX-shFST, the envelope plasmid pMD2G and the packaging plasmid psPAX2 were co-transfected into 293T cells. Lentiviral supernatants were harvested through a 0.45 μm filter 48 hours after transfection. Equal amount of Lentiviral supernatants were used for knockdown of FST in cultured cells and in mouse lung tissue.

### Mouse treatment

Male ICR mice of six weeks of age were used for the study. Mice were instilled intratracheally with PBS or 100 μg of SiO_2_ NPs for indicated time and then sacrificed^18^. Mice lung tissue was fixed in 10% formalin or stored in liquid nitrogen for mRNA and protein extraction. All studies were performed following the ethical guidelines of the Ethics Committee of Laboratory Animal Care and Welfare, Zhejiang University, School of Medicine. All studies were approved by the Ethics Committee of Laboratory Animal Care and Welfare, Zhejiang University, School of Medicine.

### Terminal deoxynucleotidyl transferase dUTP nick end labeling (TUNEL) assay

TUNEL assays were performed using the TUNEL assay kit (Promega, Madison, WI, United States) to assess cell death following the manufacture’s instruction. Localized green fluorescence of apoptotic tissue was detected with the Nikon A1R confocal microscope (Nikon Instruments Inc., Shanghai, China).

### Chromatin immunoprecipitation (ChIP)

ChIP assays were performed using the ChIP assay kit (Thermo Fisher Scientific) following the manufacturer’s protocol. Briefly, A549 cells or mouse lung tissues were cross-linked with 1% formaldehyde for 10 min at 37 °C. Cross-linking was stopped with 0.125 M glycine. For tissue ChIP, mouse lung tissue was grounded and the cells were resuspended in lysis buffer. After sonication to yield DNA fragments of 300–1000 base pairs, the lysates were cleared by centrifugation, diluted 6-fold with ChIP dilution buffer, and precleared with salmon sperm DNA/protein A-agarose at 4 °C for 1 h. For each immunoprecipitation assay, the lysates were incubated with 2 μg of anti-K4-dimethylated histone H3 (H3K4me2) (Thermo Fisher Scientific), anti-acetylated histone H3 (Lys9/18) (Ac-H3, K9/18) (Thermo Fisher Scientific), Nrf2 (Abcam, Cambridge, MA, United States) or control IgGs (Santa Cruz Biotechnology) overnight at 4 °C with rotation. The immunecomplexes were then collected with protein A-agarose slurry, eluted, and de-crosslinked at 65 °C. After RNase digestion and proteinase digestion, immunoprecipitated DNA was extracted. The purified DNA was amplified by real time PCR with SYBR GREEN PCR Master Mix (Applied Biosystems).

Sequences of the primers used for FST promoter amplification are listed in supplemental Table 1.

### Statistical analysis

The experiments were repeated at least three times and data were presented as mean ± SD. Statistical significance between two groups was determined with the Student’s *t*-test. Statistical significance among more than 3 groups was determined with one-way analysis of variance (ANOVA). All comparisons were two-tailed and *P* < 0.05 was considered significant.

## Additional Information

**How to cite this article**: Lin, C. *et al.* Transcriptional activation of follistatin by Nrf2 protects pulmonary epithelial cells against silica nanoparticle-induced oxidative stress. *Sci. Rep.* **6**, 21133; doi: 10.1038/srep21133 (2016).

## Supplementary Material

Supplementary Dataset 1

## Figures and Tables

**Table 1:** Hydrodynamic size, zeta potential, and polydispersity index of SiO2 NPs in RPMI 1640 at different time points.

Time (hour)	Hydrodynamic size (nm)	Zeta potential (mV)	Polydispersity index
0	41.36 ± 2.55	−33.28 ± 0.65	0.18 ± 0.02
24	41.32 ± 3.19	−35.14 ± 0.71	0.19 ± 0.03
