# Supplementary material for: Transcriptional activation of follistatin by Nrf2 protects pulmonary epithelial cells against silica nanoparticle-induced oxidative stress
Source: Sci Rep. 2016 Feb 16;6:21133. doi: 10.1038/srep21133 (PMC4754796; doi:10.1038/srep21133)
Supplement: Supplementary Dataset 1 [file srep21133-s1.doc]

**Transcriptional activation of follistatin by Nrf2 protects pulmonary epithelial cells against silica nanoparticle-induced oxidative stress**

Chen Lin^1#^, Xinyuan Zhao^1#^, Desen Sun^1,3^, Lingda Zhang^1^, Wenpan Fang^1^, Tingjia Zhu^1^, Qiang Wang^1^, Botao Liu^4^, Saisai Wei^3^, Guangdi Chen^1,3^, Zhengping Xu^1,2,3,*^, Xiangwei Gao^1,3,*^.

^1^Institute of Environmental Medicine, Zhejiang University School of Medicine, Hangzhou, China.

^2^Collaborative Innovation Center for Diagnosis and Treatment of Infectious Diseases, Zhejiang University, Hangzhou, China.

^3^Program in Molecular Cell Biology, Zhejiang University School of Medicine, Hangzhou, China.

^4^University of Massachusetts Medical School, Program in Molecular Medicine, Worcester, MA, 01605, USA

* To whom correspondence should be addressed: Xiangwei Gao, Ph,D. Tel: +86-571-88208169; Fax: +86-571-88208169; E-mail: xiangweigao@zju.edu.cn. Institute of Environmental Medicine, Zhejiang University School of Medicine, 866 Yuhangtang Road, Hangzhou 310058, China.

* Correspondence may also be addressed to: Zhengping Xu, Ph.D. Tel: +86-571-88208008; Fax: +86-571-88208008; E-mail: zpxu@zju.edu.cn. Institute of Environmental Medicine, Zhejiang University School of Medicine, 866 Yuhangtang Road, Hangzhou 310058, China.

# These authors contributed equally to the work.

**SUPPLEMENTARY INFORMATION**

**Supplementary Figures**

**Supplementary Tables**

**Supplementary figures.**

**Figure S1. 50 μg of SiO_2_ NPs up-regulates FST mRNA level.**

**
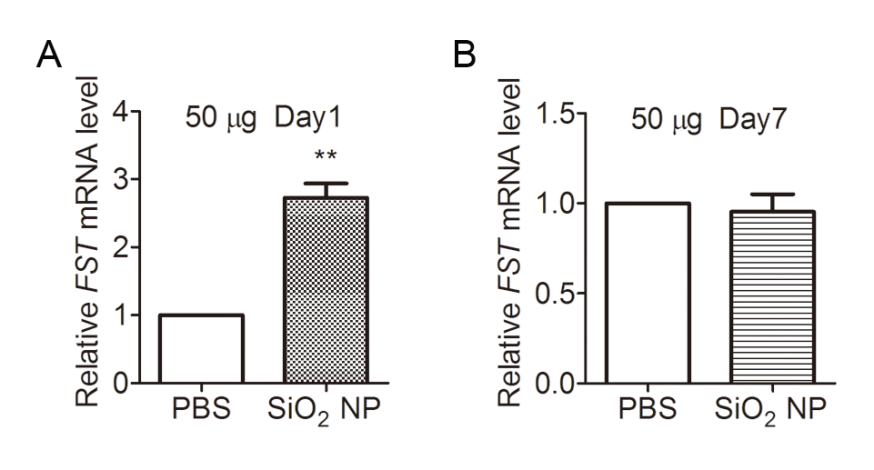
**

Mouse lung was instilled with PBS or 50 μg of SiO2 NPs and harvested at day1 (A) and day7 (B). The FST mRNA level was measured by real time qPCR and normalized to *β-actin* gene.

**Figure S2. SiO_2_ NP enhances FST expression in BEAS-2B cells.**

**
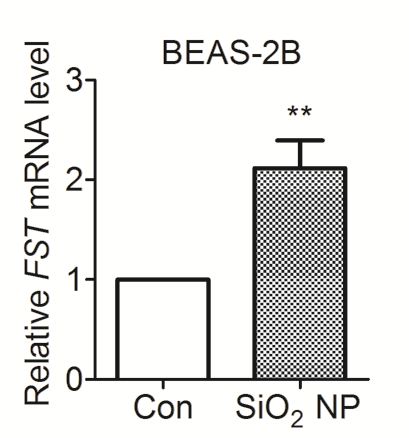
**

BEAS-2B cells were incubated with or without 50 μg/ml of SiO_2_ NPs for 12 h. The FST mRNA level was measured by real time qPCR.

**Figure S3. Actinomycin D blocks SiO_2_ NP-induced FST expression.**


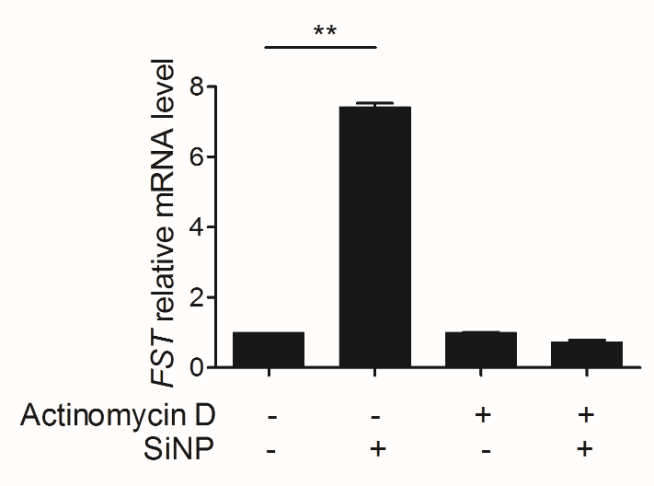


A549 cells pretreated with 5 μg/ml of actinomycin D for 1 h were incubated with or without 50 μg/ml SiO_2_ NP for another 12 h. Relative FST mRNA level was detected by real time qPCR.

**Figure S4. The effect of TiO2 nanoparticle, graphene oxide (GO), gold nanorods (GNRs), ZnO nanoparticle, or Ag nanoparticle on FST mRNA expression.**


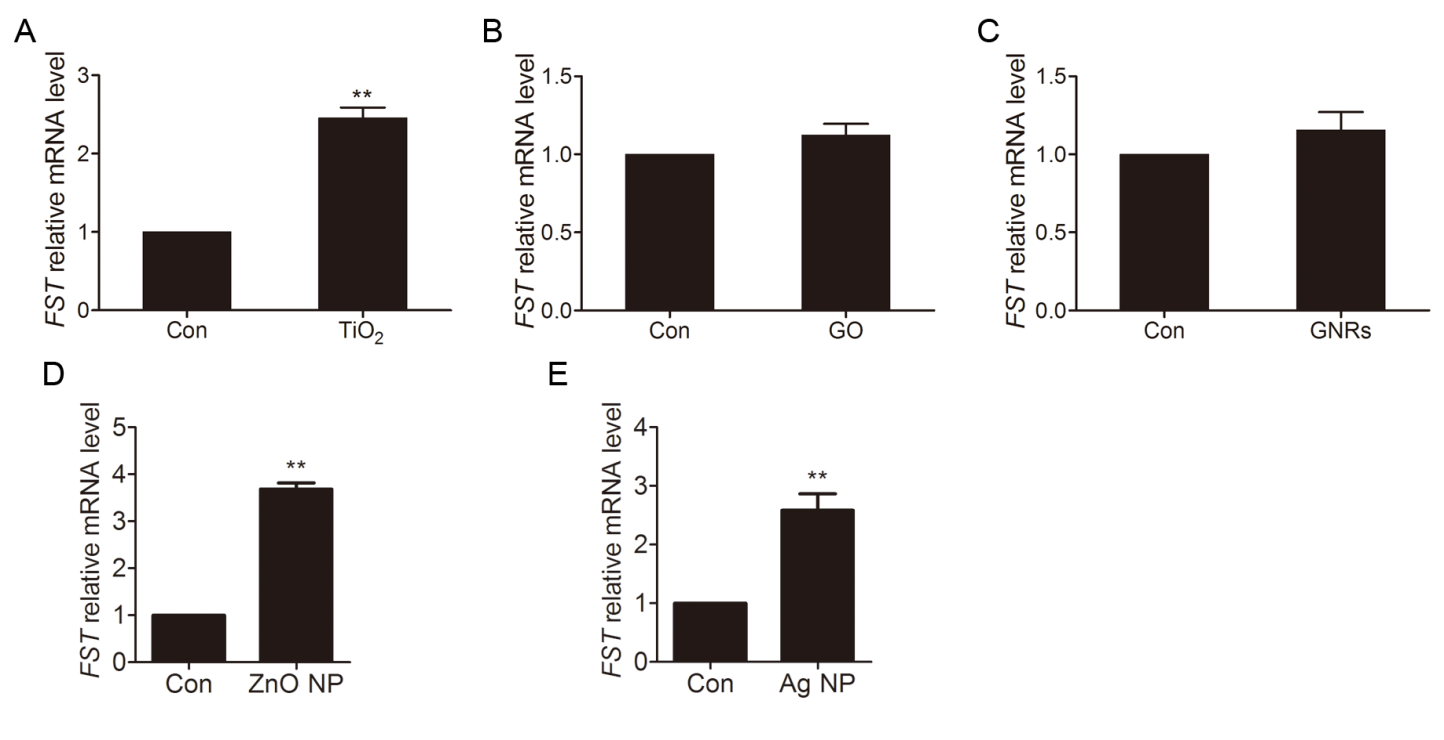


A549 cells were incubated with 50 μg/ml of TiO2 nanoparticle (A), 50 μg/ml of graphene oxide (B), 50 μg/ml of gold nanorods (C), 5 μg/ml of ZnO nanoparticle (D), or 50 μg/ml of Ag nanoparticle (E) for 12 h. The FST mRNA level was measured with real time qPCR.

**Supplemental table 1.**

Primers used in this study.

| **Gene name** | **Primer name** | **Sequence** |
| --- | --- | --- |
| Human *β-actin* | H-ACTB-F | 5’-CACGATGGAGGGGCCGGACTCATC-3’ |
|  | H-ACTB-R | 5’-TAAAGACCTCTATGCCAACACAGT-3’ |
| Human *FST* | H-FST-F | 5’-AGTCCTGTGAAGATATCCAGTGC-3’ |
|  | H-FST-R | 5’-ACTTACTGTCAGGGCACAGC-3’ |
| Human *FST* promoter | H-FSTP-F | 5’-GACTAGTCCAGACGAAGGGC-3’ |
|  | H-FSTP-R | 5’-GTGAGTGCCTGACTTTGCAG-3’ |
| Mouse *β-actin* | Mβ-actin-F | 5’-GCAGATGTGGATCAGCAAGC-3’ |
|  | Mβ-actin-R | 5’-AGCTCAGTAACAGTCCGCC-3’ |
| Human *FST* promoter2 | H-FSTP2-F | 5’-GAGGTCACCCGCTTTTACCA-3’ |
|  | H-FSTP2-R | 5’-GGCATGAATCACAAAGCCCC-3’ |
| *FST* promoter cloning | H-FSTP3-F | 5’-CCGCTCGAGGAATGAGTTGGAGGACTGGAG-3’ |
|  | H-FSTP3-R | 5’-CCCAAGCTTCAATTATCTTTCGGAGGTGC-3’ |
| Mouse *FST* | M-FST-F | 5’-AGTAAGTCGGATGAGCCGGT-3’ |
|  | M-FST-R | 5’-TTCACTTCAAGAAGCACGCC-3’ |
| Mouse *FST* promoter | M-FSTP-F | 5’-GGCTAGAGAAGAAGGGCGAA-3’ |
|  | M-FSTP-R | 5’-CTTCTGAAGCCCGAACGACA-3’ |
| Human *NOX5* | H-NOX5-F | 5’-TGTTCATCTGCTCCAGTTCC-3’ |
|  | H-NOX5-R | 5’-ACAAGATTCCAGGCACCAG-3’ |
| Human *NOX4* | H-NOX4-F | 5’-TCACAGAAGGTTCCAAGCAG-3’ |
|  | H-NOX4-R | 5’-ACTGAGAAGTTGAGGGCATTC-3’ |
| Human *NOX3* | H-NOX3-F | 5’-TGAGGGTCTCTCCACCATATT-3’ |
|  | H-NOX3-R | 5’-ACTCCTCCTCTTCATACCAGTAG-3’ |
| Human *NOX2* | H-NOX2-F | 5’-CCAGTGAAGATGTGTTCAGCT-3’ |
|  | H-NOX2-R | 5’-GCACAGCCAGTAGAAGTAGAT-3’ |
| Human *NOX1* | H-NOX1-F | 5’-GCAAATGCTGTCACCGATATTC-3’ |
|  | H-NOX1-R | 5’-TGCAGATTACCGTCCTTATTCC-3’ |
| Human *Nrf2* | H-Nrf2-F | 5’-TGCCCCTGGAAGTGTCAAAC-3’ |
|  | H-Nrf2-R | 5’-CCCCTGAGATGGTGACAAGG-3’ |
| Mouse *FST* shRNA | M-FST-S | 5’-gatccGTGTGACCTGTAATCGGATTTCAAGAGAATCCGATTACAGGTCACACTTTTTTg-3’ |
|  | M-FST-AS | 5’-aattcAAAAAAGTGTGACCTGTAATCGGATTCTCTTGAAATCCGATTACAGGTCACACg-3’ |
